# Prioritization: Run This Board: Septic Shock, Acute Coronary Syndrome, Small Bowel Obstruction, and Penetrating Chest Trauma

**DOI:** 10.21980/J8.52355

**Published:** 2025-12-31

**Authors:** Colleen Donovan, Nicole Novotny, Charles Lei, Alaa Aldalati, Andrew Melendez, Neil Wallace, Tiffany Moadel, Stephanie Stapleton, Shagun Berry

**Affiliations:** 1 Rutgers Robert Wood Johnson Medical School, Department of Emergency Medicine, New Brunswick, NJ; 2 Ochnser Health System, Department of Emergency Medicine, New Orleans, LA; 3 Hennepin County Medical Center, Department of Emergency Medicine, Minneapolis, MN; 4 University of Kansas School of Medicine-Wichita, Wesley Medical Center Emergency Department, Wichita, KS; 5 Yale University, Department of Emergency Medicine, New Haven, CT; 6 University of Arizona/Banner Medical Center, Department of Emergency Medicine, Phoenix, AZ; 7 Zucker School of Medicine at Hofstra/Northwell, Department of Emergency Medicine, Hempstead, NY; 8 Boston University/Boston Medical Center, Department of Emergency Medicine, Boston, MA; 9 Rush University Medical Center, Department of Emergency Medicine, Chicago, IL

## Abstract

**Audience:**

This case was specifically designed for senior emergency medicine (EM) resident physicians as a preparatory tool for the American Board of Emergency Medicine (ABEM) Certifying Exam. However, it is applicable for EM residents at all levels of training.

**Introduction:**

“A hallmark of emergency medicine is the ability to triage or prioritize care. This case will require the physician to evaluate and treat multiple patients while ensuring those who require immediate care receive it quickly. The physician may face the arrival of additional patients, the deterioration of existing patients, and realistic workflow interruptions during the case. A successful candidate will identify and stabilize high acuity patients.”^1^ With the introduction of the new Certifying Exam by ABEM and the current lack of resources to practice prioritization in an assessment setting, resident physicians will need practice material in order to adequately prepare for their board examination.

**Educational Objectives:**

By the end of this case learners will be able to: 1) Become familiar with format of a prioritization case (a component of the ABEM Certifying Exam), 2) Practice their ability to prioritize multiple 313 patients and provide stabilizing care, 3) Consider changes in status/patient acuity/new cases as presented, 4) Understand how to utilize team resources appropriately.

**Educational Methods:**

This encounter is a structured, oral simulation case designed as a prioritization exercise for emergency medicine resident trainees. It follows an interview-based format in which an examiner presents evolving patient information in a time-limited scenario, and the examinee responds in real time with clinical reasoning, prioritization of care, and management decisions. The case mimics the structure of the Prioritization Case in the ABEM Certifying Exam, allowing the examinee to verbalize thought processes while receiving updated clinical data from the examiner. This format emphasizes critical thinking, triage under pressure, and the delegation of tasks within a simulated emergency department environment.

**Research Methods:**

We first alpha-tested the case with board-certified emergency medicine physicians, who evaluated both the facilitator and learner aspects of the simulation. Their feedback was used to refine the structure, flow, and clinical realism of the case. Following these edits, the revised case was implemented with emergency medicine residents across varying levels of training. This staged approach allowed us to ensure educational fidelity and enhance realism, while also confirming that the case structure aligned with ABEM exam standards and expectations.

**Results:**

We conducted multi-site alpha and beta testing of a novel ABEM-style prioritization case with a total of 18 emergency medicine residents (eight individual residents and two 5-person PGY2 teams) and three facilitators. Surveys were completed by two facilitators (Simulation Scenario Evaluation Tool, SSET) and eight resident participants or teams (modified usability survey). Facilitators rated the case highly, with an average global score of 87.5/100, and learners reported strong overall quality (4.4/5) and exceptional educational value (4.9/5), though clarity of instructions was rated lower (3.6/5). Participants were predominantly senior residents (62.5% PGY-3, 25.0% PGY-2, and 12.5% PGY-4). Qualitative comments emphasized the usefulness of practicing the new case format and highlighted a need for clearer explanations of structure and rules.

These results suggest the case was well-received across training levels, with iterative revisions improving clarity and usability. Based on preliminary beta testing, a Total Score of 70–75% indicates passing for this case.

**Discussion:**

This oral board-style prioritization case offers learners the opportunity to practice essential but often underemphasized skills, including rapid prioritization, task switching, and real-time decision-making. The case format reinforces critical concepts such as situational awareness and resource management within the dynamic environment of the emergency department. During initial implementation, participants reported strong engagement and found the exercise particularly valuable in preparing for the ABEM Certifying Exam. Many noted that the structure and expectations closely mirrored those of the actual prioritization station based on the example video provided by ABEM.

From the instructor perspective, the standardized format promotes consistent delivery and assessment. To minimize examiner cognitive load during this high-complexity simulation, we developed a modular toolkit including a structured script, stimuli slides, and an automated scoring sheet, modeled in part after ABEM’s dual-examiner approach. A suite of Appendices supports both digital and paper-based use, allowing flexibility across educational settings.

To further support formative practice, we created a scoring rubric to guide examiner feedback and learner self-assessment. However, as ABEM has not released its internal scoring criteria, this rubric is unofficial and should be interpreted with caution. It is intended for educational use only and is not designed to predict performance on the actual certification exam.

**Topics:**

Prioritization, triage, stabilization, delegation, task switching, vaginal bleeding, pediatric injury, altered mental status, septic shock, acute coronary syndrome, headache, abdominal pain, small bowel obstruction, penetrating chest trauma, urinary tract infection.

## USER GUIDE

**Table:** 

List of Resources:	
Abstract	312
User Guide	315
For Examiner Only	319
Appendix 1: How to Set Up This Scenario	319
Appendix 2: Examiner Script with Linked Objectives	325
Appendix 3: Stimuli Slides and Stimuli to Print	340
Appendix 4: Scoring Sheet	354
Appendix 5: Blank Tracking Board for Examinees to Print	355
Appendix 6: Certifying Exam Assessment Scoring Sheet to Print	356
**Appendix 7:** Debrief Guide	363

**Learner Audience:**

This prioritization exercise is directed to interns and junior and senior residents, specifically for preparation for the ABEM Certifying Exam.

**Time Required for Implementation:**

Case: Prioritization cases are 15 min by the American Board of Emergency Medicine (ABEM) standard

Debriefing: 15–30 minutes

**Recommended number of learners per instructor:**

This case was originally designed for individual practice by one senior emergency medicine (EM) resident, supported by two instructors, to help prepare for the American Board of Emergency Medicine (ABEM) Certifying Exam. Although targeted toward senior residents, the case is appropriate for EM residents at all levels of training. Small group participation may also be beneficial, as demonstrated during our beta testing with PGY-2 residents at the SAEM conference.

While the format mirrors the ABEM Prioritization example case with two faculty facilitators, it can also be adapted for use with a single instructor, provided that person is highly familiar and confident with the case content. Additionally, we believe involving a senior resident in the second instructor role can offer valuable insight into the structure, flow of the case, and scoring, serving as an effective teaching and preparatory experience.

**Topics:**

Prioritization, triage, stabilization, delegation, task switching, vaginal bleeding, pediatric injury, altered mental status, septic shock, acute coronary syndrome, headache, abdominal pain, small bowel obstruction, penetrating chest trauma, urinary tract infection.

**Objectives:**

By the end of this case learners will be able to:

1. Become familiar with format of a prioritization case (a component of the ABEM Certifying Exam)
2. Practice their ability to prioritize multiple patients and provide stabilizing care
3. Consider changes in status/patient acuity/new cases as presented
4. Understand how to utilize team resources appropriately

### Linked objectives, methods and results

In the ABEM Certifying Exam, this clinical case is a structured discussion with two examiners. This practice case may be done with one or two examiners.

This case will assess the ability to prioritize multiple patients in the emergency department or prehospital setting and provide stabilizing care.

The successful candidate will:

- Determine acuity of patient(s)
- Provide appropriate and immediate stabilizing care
- Respond to changes in patient acuity and triage new cases as presented
- Use team resources appropriately

1. Patient Prioritization & Initial Assessment:
  - Analyze a patient tracking board and determine the most critical patients based on limited initial information.
  - Identify key history, physical exam findings, and immediate diagnostic tests needed for rapid decision-making.
2. Stabilization & Initial Management:
  - Implement appropriate immediate interventions for critically ill patients, such as noninvasive ventilation, fluid resuscitation, or procedural sedation.
  - Recognize and manage time-sensitive conditions, including airway compromise, hemodynamic instability, and altered mental status.
3. Task Delegation & Team Management:
  - Identify tasks that can be delegated to the healthcare team to optimize workflow and efficiency.
  - Communicate clear and concise instructions to supporting staff during patient management.
4. Dynamic Adaptation & Task Switching:
  - Adjust clinical priorities based on additional diagnostic data and evolving patient conditions.
  - Reprioritize care as new patients arrive, balancing initial assessments with ongoing interventions.
5. Procedural Decision-Making & Execution:
  - Determine the appropriate use of sedation and procedural interventions for acutely ill patients.
  - Perform or direct critical procedures such as intubation, fracture reduction, and vascular access placement.
6. Case Completion & Reflection (Debrief):
7. Review and reflect on decision-making strategies at the conclusion of the case.
  - Identify areas for improvement in prioritization, delegation, and procedural execution.

### Recommended pre-reading for instructor

- Instructors should watch the ABEM Prioritization sample case video here, and be very familiar with all of the included Appendices: Appendix 1. How to set up this scenario, Appendix 2. Examiner Script with Linked Objectives, Appendix 3. Stimuli Slides and Stimuli (To Print), Appendix 4. Score Sheet.

### Scenario Materials and Appendices

Due to the complexity and cognitive demands of the Prioritization Case format, we intentionally deviated from traditional manuscript structure to prioritize clarity, usability, and replicability. This case was designed and implemented using a modular system of linked materials: a facilitator script (Word/Google Docs), stimuli slides (PowerPoint/Google Slides), and an automated scoring spreadsheet (Excel/Google Sheets). These tools were created to minimize facilitator cognitive load during gameplay, allowing greater focus on examinee performance and formative feedback. Notably, the American Board of Emergency Medicine (ABEM) appears to assign two examiners to each Prioritization Case to help manage the volume of information, highlighting the importance of streamlining cognitive demands for facilitators.

To support flexible implementation across various educational environments, we have included a suite of Appendices. These include digital and print-ready resources that guide scenario setup, case delivery, scoring, and debriefing. Additionally, printable PDFs are provided for users who prefer to run the case without digital components. Detailed instructions and alternative workflows are outlined in Appendix 1. We believe this approach enhances fidelity to the real-world exam experience while improving facilitator efficiency and educational impact.

#### Appendix 1. *How to Set Up This Scenario “Run This Board…” Prioritization Case*

- Step-by-step setup guide

#### Appendix 2. *Examiner Script with Linked Objectives*

- Full facilitator script with Tracking Boards, stimuli prompts, and objective alignment

#### Appendix 3. *Stimuli Slides and Stimuli (To Print)*

- All stimuli, including Tracking Boards, hyperlinked images, and case references

#### Appendix 4. *Scoring Sheet*

- Spreadsheet file with automated scoring across the following domains:
  ○ 25% Prioritization Questions (Tab 1)
  ○ 25% Stabilization Actions (Tab 1)
  ○ 12.5% Delegation Actions (Tab 1)
  ○ 12.5% Task Switching Actions (Tab 1)
  ○ 25% Prioritization Order (Tab 2)

#### Appendix 5. BLANK Tracking Board for Examinees (To Print)

- Printable Tracking Board template for examinee use

#### Appendix 6. Scoring Sheet (To Print)

- Printable scoring templates for manual scoring

#### Appendix 7. *Debrief Guide*

- A Facilitator Debrief Script with additional instructions on hand scoring, if needed

### Results and tips for successful implementation

We employed an iterative trial process across multiple sites using a convenience sample of emergency medicine residents. Feedback on the experience was gathered from both facilitators and learners through anonymous surveys, which included Likert-scale items (ranging from 1 = strongly disagree to 5 = strongly agree) and optional open-ended responses. Survey data were collected using Qualtrics^3^ and analyzed in Microsoft Excel.^4^ This study was reviewed by the Boston University Institutional Review Board and determined to be exempt from further oversight.

We tested the case on seven individual learners (six PGY 3, one PGY 4), two small group resident teams (five PGY 2 residents per group) and three facilitators.

We employed an iterative, multi-site case testing process using a convenience sample of emergency medicine physicians. Alpha testing was conducted at a separate academic institution, where a facilitator piloted the case and completed the Simulation Scenario Evaluation Tool (SSET) survey^5^ to assess the quality of core simulation elements.

First round beta testing was conducted at a different, separate academic institution, where a facilitator piloted the case with their EM residents. The facilitator completed the SSET survey, while residents provided feedback through a modified usability survey.

Second round beta testing was conducted at the Society for Academic Emergency Medicine (SAEM) Annual Meeting in May 2025, Philadelphia, PA, where two small groups of five PGY-2 residents each participated in the case and completed the same usability survey. Each group completed the modified usability survey as a team.

Third round beta testing took place at a third academic medical center with one facilitator and five senior EM residents, four of whom completed the modified usability survey. Feedback from all phases was used to iteratively refine the case.

A total of two facilitators completed the SSET survey and eight residents or small group resident teams filled out the modified usability survey.

Facilitator feedback was collected across three completed reviews using the SSET and a global rating scale. Overall, the case was rated highly, with an average global score of 87.5 out of 100, indicating strong perceived quality and usability. Reviewers consistently rated the simulation’s learning objectives, scenario materials, and patient states favorably. This feedback supported iterative improvements to the case structure, content clarity, and debriefing materials throughout the trialing process.

A total of eight emergency medicine residents or resident teams completed the learner usability survey following beta testing of the prioritization case. The majority of respondents were PGY-3 residents (62.5%), followed by PGY-2s (25.0%), and PGY-4s (12.5%).

Participants rated the case highly across three key domains:

- **Overall quality**: Average rating of 4.4/5 (88%)
- **Clarity of instructions**: Average rating of 3.6/5 (72%)
- **Educational value**: Average rating of 4.9/5 (98%)

These results indicate that learners found the case to be highly educational and well-constructed, though there is some variability in perceptions of instructional clarity.

### Sample Positive Feedback

*"It was helpful to experience the new format and run through a case. It is a completely different format from any exams I have taken, so the more samples I go through, the better."*

### Sample Constructive Feedback

*"More clarifying points [regarding structure and rules.]"*

### Advice for Facilitators

- Refer to Appendix 1 for detailed instructions on setting up the scenario.
- Learners expressed uncertainty about what could be ordered at the beginning of the case. Clarify whether “1–2 pieces of information” refers to history, physical exam findings, labs, or imaging.
- Clarify to learners that they should ask initial questions per patient, not just 1–2 questions total across all cases during the initial prompt.
- Although the examiner in the sample video prompts for only 1–2 responses, the examinee often asks 3–5 or more pieces of information. While the exact scoring rubric for the exam is not publicly available, we operated under the assumption that the examinee in the video demonstrated a passing performance. Accordingly, we evaluated our participants using a weighted scoring system based on similar response patterns.
- Remind learners that there are two nurses available for task delegation. Encourage them to utilize these team members and remember it’s possible to use them more than once.
- Do not share diagnostic stimuli (eg, EKGs, chest X-rays) until after the learners have selected that specific patient to prioritize. For example, do not show Stimuli 3 & 4 until the learners have decided to evaluate Patient D. Learners should not have access to all stimuli at once to decide which room to assess first. Giving stimuli early will interfere with accurate scoring.
- During beta testing, some learners focused on disposition with the intent to discharge and simplify their tracking board. Facilitators should reinforce that the objective is stabilization and prioritization, not disposition or discharge planning.

### Advice for Residents/Examinees

- At the opening of the case, although the examiner in the sample video prompts for only “1–2 pieces of information,” we encourage you to gather 3–5 pieces of information rather than the 1–2 pieces suggested in the prompt. This advice is based on the performance of the passing examinee shown in the example video.
- When the examiner prompts the examinee for 1–2 pieces of information, one could reasonably argue for including a SAMPLE history (based on common prehospital reporting formats) and a primary survey. That said, we believe it is unlikely that ABEM examiners will allow this approach on the actual exam and thus recommend the above advice.
- Use your available team resources, such as nurses, to help with triage, data gathering, and interventions.
- Be prepared to justify your prioritization based on clinical reasoning, not just test results or imaging.
- If unsure what you are allowed to request initially (history, physical, labs), ask for clarification or state your assumptions out loud.
- Focus on clinical stabilization and prioritization rather than trying to disposition or discharge patients during the case.

### Scoring Rubric Disclaimer

The scoring rubric included with this simulation was developed to provide examinees and facilitators with a structured framework for practicing the ABEM Prioritization Case format. Given the high-stakes nature of the ABEM Certifying Exam and the lack of publicly available scoring criteria, we aimed to create a practical tool to support formative assessment, examiner calibration, and personalized improvement plans. However, we acknowledge that we do not have access to ABEM’s official scoring methodology.

According to ABEM’s *A Guide to ABEM Certification Webinar for Graduating EM Residents*, presented on June 3, 2025, the Board does not intend to release example scoring rubrics due to concerns that doing so may compromise the validity of the examination (covered at timestamp 47:25).^2^ Furthermore, while the video confirms that each Prioritization Case is evaluated by two examiners, no additional details on scoring standards or weighting are provided.

Because this new component of the exam is scheduled to launch in Spring 2026, less than a year from the writing of this manuscript, we felt it was important to offer an educational resource that programs could begin using immediately. In doing so, we acknowledge that we have prioritized timely dissemination over formal validation in order to meet the urgent need for preparation materials.

As such, our rubric represents a best-faith approximation based on expert consensus, face validity, and the published exam structure, but it may differ substantially from the actual scoring used by ABEM. Facilitators and examinees should interpret the results accordingly and understand that this rubric is intended for educational purposes only. It should not be used to predict or guarantee performance on the actual certification exam.

## Figures and Tables

**Figure:**
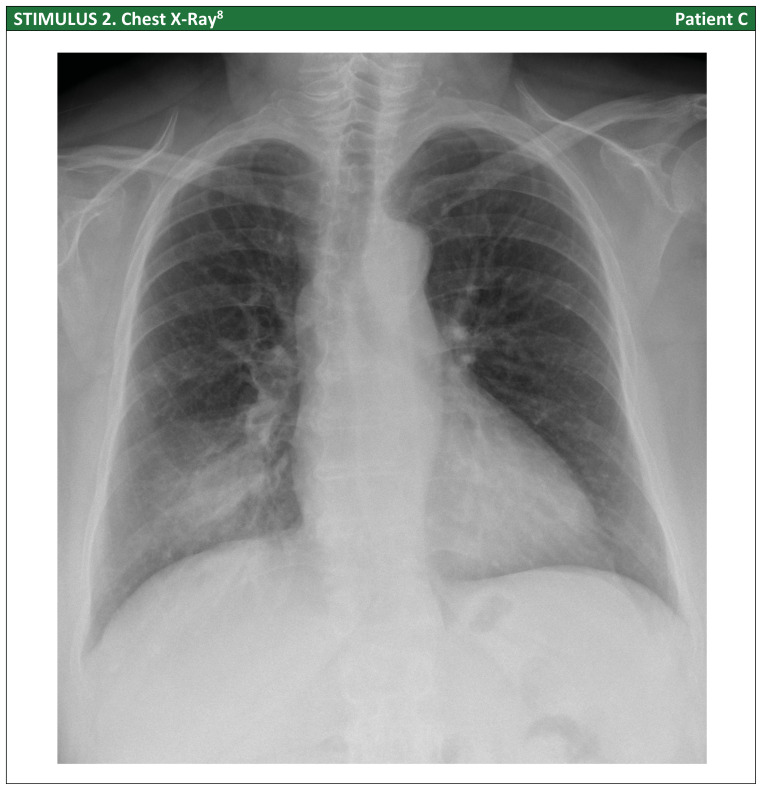
Please see associated PowerPoint file

**STIMULUS 1 f4-jetem-10-5-ce312:**
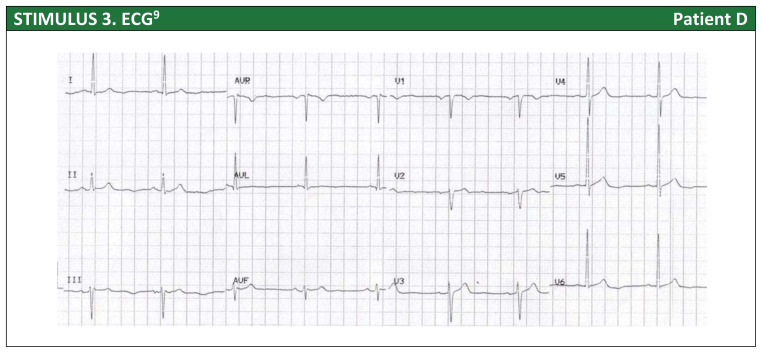
ECG^7^ Patient C

**STIMULUS 2 f5-jetem-10-5-ce312:**
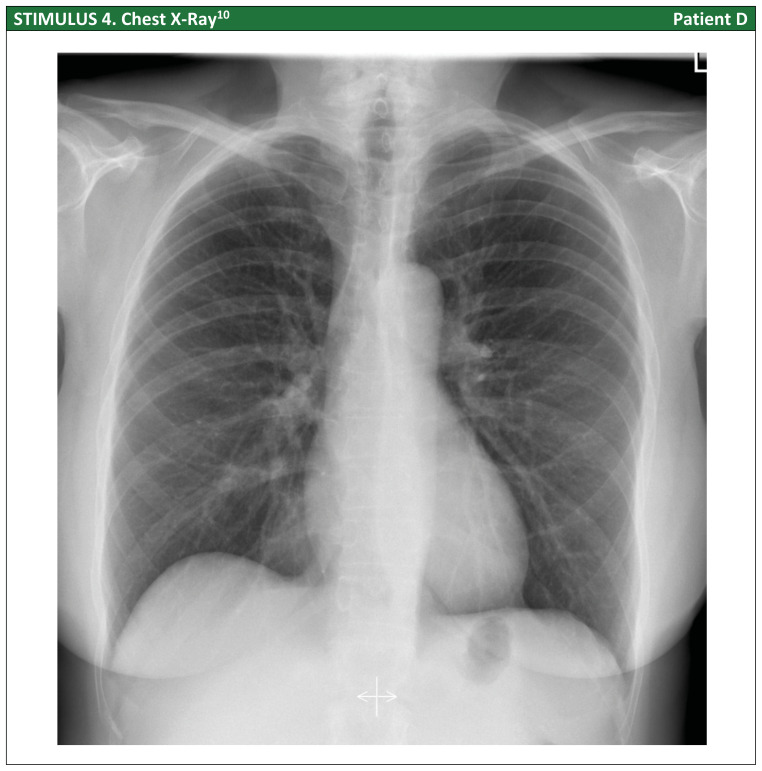
Chest X-Ray^8^ Patient C

**STIMULUS 3 f6-jetem-10-5-ce312:**
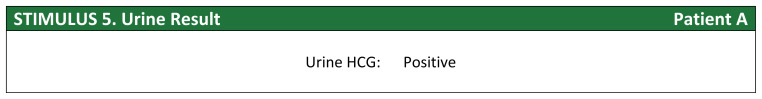
ECG^9^ Patient D

**STIMULUS 4 f7-jetem-10-5-ce312:**
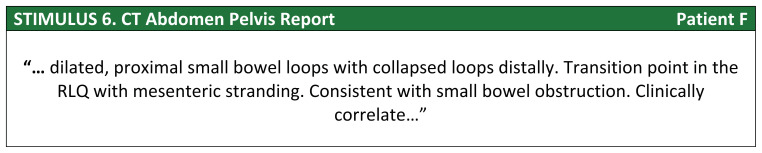
Chest X-Ray^10^ Patient D

**Figure:**
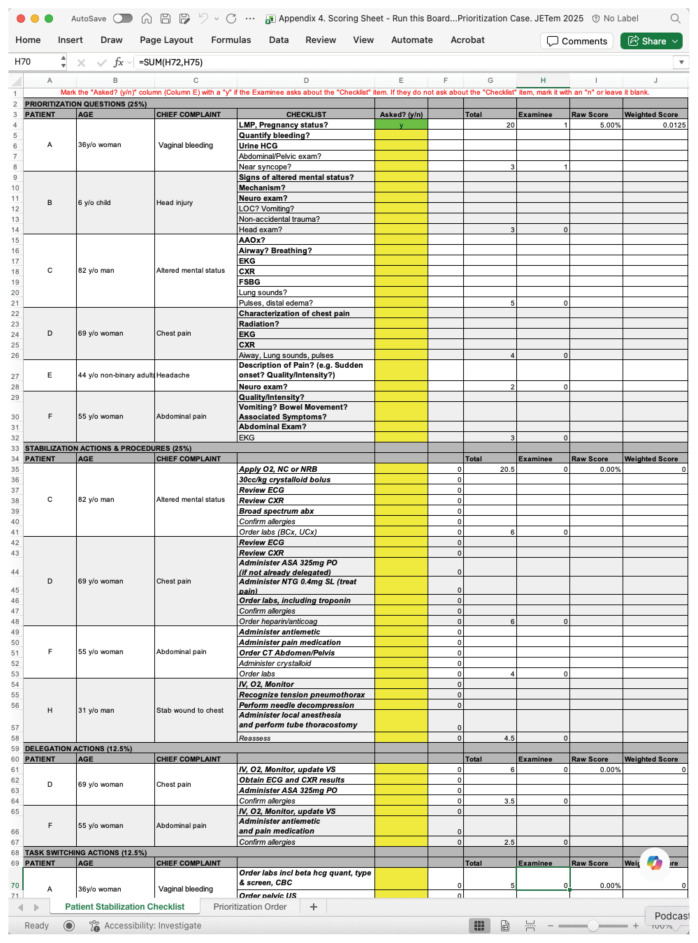
Please see associated Excel file

## FOR EXAMINER ONLY

### Appendix 1 Prioritization: Run This Board How to Set Up This Scenario

Please be sure to watch the ABEM Prioritization sample case video here.

The “Run this Board…” Prioritization Appendices contain 7 files:

- Appendix 1. How to set up this scenario - “Run this Board…” Prioritization Case
  ○ Step by step set up instructions
- Appendix 2. Examiner Script with Linked Objectives
  ○ Full script with Tracking Boards and stimuli prompts
- Appendix 3. Stimuli Slides and Stimuli (To Print)
  ○ Slides with Tracking Boards, hyperlinked stimuli and references
- Appendix 4. Scoring Sheet
  ○ Spreadsheet for automatic scoring
- Appendix 5. BLANK Tracking Board for Examinees (To Print)
  ○ Printable Tracking Board template for examinee use
- Appendix 6. Scoring Sheet (To Print)
  ○ Printable scoring templates for manual scoring
- Appendix 7. Debrief Guide
  ○ Suggested debrief guide with additional instructions on hand scoring, if needed

There are 3 main ways to run this scenario, with hybrid combinations in-between.

- Two Computer Setup
- Dual Extended Monitor Setup
- Printed Paper Setup
- Other Hybrid Options

**Two Computer Setup:**

This setup is entirely computer based.

Benefits include easy, real-time scoring.

Drawbacks include the need for 2 computers and some computer setup skills.

Step-by-step instructions:

- Download the “Run this Board…” Prioritization Case folder on Computer 1 and Computer 2.
- Open Appendix 2. Examiner Script with Linked Objectives - “Run this Board…” Prioritization Case and Appendix 4. Scoring Sheet - “Run this Board…” Prioritization Case on Computer 1.
  ○ In Windows:
    With Appendix 2. Examiner Script on top, hover over the top right “maximize” button (square) and choose the side-by-side panel with Appendix 2. Examiner Script on left side of the screen and Appendix 4. Scoring Sheet on the right side of the screen.
  ○ In iOS:
    With Appendix 2. Examiner Script on top, hover over the top left “maximize/zoom” button (green circle) and choose the side-by-side panel with Appendix 2. Examiner Script on left side of the screen and Appendix 4. Scoring Sheet on the right side of the screen.
- Open Appendix 3. Stimuli Slides PPT - “Run this Board…” Prioritization Case Slides on Computer 2.
  ○ If using Google Slides, start Slideshow.
    ▪ Don’t use Presenter View unless you want a lot of widows open.
  ○ If using PowerPoint, go to Slide Show → Set Up Slide Show → Browsed by individual (window) → OK.
    ▪ This will prevent Presenter View from opening and only show the slides on Computer 2, so you don’t have to deal with extra open windows.
- Print one Appendix 5. BLANK Tracking Board for Examinees - “Run this Board…” Prioritization Case for each of your Examinees.

You are now ready as the Examiner to read the script on left Display 1, score in real time on right Display 1, and show stimuli to the Examinee on Display 2.

**Dual Extended Monitor Setup:**

This setup is entirely computer based.

Benefits include easy, real-time scoring, and control from a single computer.

Drawbacks include the need for 2 monitors and some computer setup skills.

Step-by-step instructions:

- Download the “Run this Board…” Prioritization Case Appendix.
- Create your dual monitor setup.
  ○ Connect a second monitor to your computer using USB-C or HDMI.
  ○ Go to Settings on your computer.
  ○ In Windows, go to System. In iOS (Mac) go to System Preferences.
  ○ Choose Display, change “Duplicate these displays” to “Extend these displays.”
  ○ Choose Keep changes.
    ▪ Now Display 1 should be your computer and Display 2 should be your extra monitor.
- Open Appendix 3. Stimuli Slides PPT - “Run this Board…” Prioritization Case Slides on Display 2.
  ○ If using Google Slides, start Slideshow.
    ▪ Don’t use Presenter View unless you want a lot of widows open.
  ○ If using PowerPoint, go to Slide Show → Set Up Slide Show → Browsed by individual (window) → OK.
    ▪ This will prevent Presenter View from opening and only show the slides on Display 2, so you don’t have to deal with extra open windows.
- Open Appendix 2. Examiner Script with Linked Objectives - “Run this Board…” Prioritization Case and Appendix 4. Scoring Sheet - “Run this Board…” Prioritization Case on Display 1.
  ○ In Windows:
    With Appendix 2. Examiner Script on top, hover over the top right “maximize” button (square) and choose the side-by-side panel, with Appendix 2. Examiner Script on left side of the screen and Appendix 4. Scoring Sheet on the right side of the screen.
  ○ In iOS:
    With Appendix 2. Examiner Script on top, hover over the top left “maximize/zoom” button (green circle) and choose the side-by-side panel, with Appendix 2. Examiner Script on left side of the screen and Appendix 4. Scoring Sheet on the right side of the screen.
- Print one Appendix 5. BLANK Tracking Board for Examinees - “Run this Board…” Prioritization Case for each of your Examinees.

You are now ready as the Examiner to read the script on left Display 1, score in real time on right Display 1, and show stimuli to the Examinee on Display 2.

**Printed Paper Setup:**

- Print the following files from the “Run this Board…” Prioritization Case Appendix.
  ○ Appendix 2. Examiner Script with Linked Objectives - “Run this Board…” Prioritization Case.
    ▪ Your script for the case - don’t show to the Examinee.
  ○ Appendix 3. Stimuli Slides PPT - “Run this Board…” Prioritization Case Slides.
    ▪ Show one at a time to the Examinee as indicated in the Examiner Script.
    ▪ If you decide to use printed stimuli, be sure to reorder the paper slides appropriately; otherwise, they will be out of order for your Examinee.
  ○ Appendix 5. BLANK Tracking Board for Examinees - “Run this Board…” Prioritization Case.
    ▪ Print one for each Examinee.
  ○ Appendix 6. Patient Stabilization & Prioritization Score Sheets PDF.
    ▪ Print to hand score and then hand calculate the final score (instructions are in the PDF).

You are now ready as the Examiner to read the printed script, score by hand (tally for full score later) and show printed stimuli to the Examinee.

**Other Hybrid Options:**

There are plenty of other hybrid options for you to choose.

For example, you can print the Examiner Script, open and score the Score Sheet on a tablet, and show the stimuli on a computer monitor. The world is your oyster. Do what works for you.

**Flow Note on Case Progression** ***:***

**Optimal flow for this case**

- Examiner provides instructions and shows Tracking Board #1
  ○ Examinee asks Prioritization Questions about each Patient A–F and Examiner logs answers on the scoring sheet
- Show Tracking Board #2
  ○ Evaluate and stabilize first patient (Patient C)
    ▪ Show Stimulus 1 and 2
  ○ Delegate nurse to start workups on 2 patients (Patients D and F) while stabilizing first patient
  ○ Evaluate and stabilize second patient (Patient D)
    ▪ Show Stimulus 3 and 4
  ○ New information available: Urine Pregnancy Positive for Patient A (Stimulus 5)
    ▪ Task switch and delegate RN to workup Patient A
- Show Tracking Board #2.1
  ○ Evaluate and stabilize third patient (Patient F)
    ▪ Show Stimulus 6
- Show Tracking Board #3: Add Patients G and H and reprioritize
  ○ Evaluate and stabilize fourth patient (Patient H)
- Prioritize the remaining patients (Patients A, E, B, G)

In the normal flow of this case, when the third patient is addressed, Patients G and H are added via Tracking Board #3.

If there is delay, and the examinee does not make it through addressing their third patient by 10 minutes, then at the 10-minute mark, Patients G and H should be introduced via Tracking Board #3.

### Appendix 2: Prioritization: Run This Board Examiner Script with Linked Objectives

In the ABEM Certifying Exam, this clinical case is a structured discussion with two examiners. This practice case may be done with one or two examiners.

This case will assess the ability to prioritize multiple patients in the emergency department or prehospital setting and provide stabilizing care.

The successful candidate will:

- Determine acuity of patient(s)
- Provide appropriate and immediate stabilizing care
- Respond to changes in patient acuity and triage new cases as presented
- Use team resources appropriately

**Flow Note on Case Progression:**

In the normal flow of this case, when the third patient is addressed, Patients G and H are added via Tracking Board #3.

If there is delay, and the examinee does not make it through addressing their third patient by 10 minutes, then at the 10-minute mark, Patients G and H should be introduced via Tracking Board #3.

Yellow Highlight indicates something the Examiner will SAY to the Examinee as a prompt in the script.

Green Highlight indicates a Stimulus (tracking board or image) that should be SHOWN to the Examinee.

#### OBJECTIVES

1. **Patient Prioritization & Initial Assessment:**
  - Analyze a patient tracking board and determine the most critical patients based on limited initial information.
  - Identify key history, physical exam findings, and immediate diagnostic tests needed for rapid decision-making.
2. **Stabilization & Initial Management:**
  - Implement appropriate immediate interventions for critically ill patients, such as noninvasive ventilation, fluid resuscitation, or procedural sedation.
  - Recognize and manage time-sensitive conditions, including airway compromise, hemodynamic instability, and altered mental status.
3. **Task Delegation & Team Management:**
  - Identify tasks that can be delegated to the healthcare team to optimize workflow and efficiency.
  - Communicate clear and concise instructions to supporting staff during patient management.
4. **Dynamic Adaptation & Task Switching:**
  - Adjust clinical priorities based on additional diagnostic data and evolving patient conditions.
  - Reprioritize care as new patients arrive, balancing initial assessments with ongoing interventions.
5. **Procedural Decision-Making & Execution:**
  - Determine the appropriate use of sedation and procedural interventions for acutely ill patients.
  - Perform or direct critical procedures such as intubation, fracture reduction, and vascular access placement.
6. **Case Completion & Reflection (Debrief):**
  - Review and reflect on decision-making strategies at the conclusion of the case.
  - Identify areas for improvement in prioritization, delegation, and procedural execution.

#### TRANSCRIPT

*On arrival, have Appendix 3: Stimuli Slides open and displayed.*

*Start on the 2nd Slide: Prioritization Candidate Task Sheet while giving the introduction.*

**Examiner(s):**

Hello doctor, come on in.

Welcome, please take a seat.

My name is Dr. [YOUR NAME] and I will be your examiner today.

This is a prioritization case. Keep in mind that while prioritizing care, patient information and diagnostic results provided by The Examiner may be very limited. I may interrupt you to move through the case. This does not reflect your performance. In the time allotted, it's possible you may not have the opportunity to completely manage every patient on your tracking board. You'll have 15 minutes to complete the case. Before we begin, do you have any questions?

*Pause for Examinee questions/response.*

**Examiner:**

The clock starts now.

*Start your timer.*

You are a single-coverage ED physician working at a community hospital.

Six (6) patients are waiting to be seen.

Here is your patient tracking board.

*Show Tracking Board #1*

After reviewing the tracking board, let me know when you are ready to proceed.

**Table:** TRACKING BOARD #1

PATIENT	AGE	CHIEF COMPLAINT	VITAL SIGNS
A	36 y/o woman	Vaginal bleeding	BP: 112/72 P: 86 R: 16 Temp: 37.2° C (98.9° F) Sat: 99% on room air
B	6 y/o child	Head injury	BP: 104/65 P: 90 R: 18 Temp: 37.0° C (98.6° F) Sat: 99% on room air
C	82 y/o man	Altered mental status	BP: 82/65 P: 125 R: 28 Temp: 39.1° C (102.3° F) Sat: 87% on room air
D	69 y/o woman	Chest pain	BP: 150/76 P: 82 R: 16 Temp: 36.6° C (97.9° F) Sat: 99% on room air
E	44 y/o non-binary adult (assigned male at birth)	Headache	BP: 118/72 P: 78 R: 14 Temp: 36.9° C (98.4° F) Sat: 99% on room air
F	55 y/o woman	Abdominal pain	BP: 145/78 P: 98 R: 14 Temp: 37.7° C (99.9° F) Sat: 98% on room air

**Examiner:**

What one or two most important pieces of additional information from history, physical exam, or immediately available diagnostic testing would you like to know about each Patient A through F in order for you to prioritize care?

- *Addresses Objective 1:* ***Patient Prioritization and Initial Assessment***

*Pause for Examinee response.*

*Record answers on the* *Scoring Sheet.*

**Table:** TRACKING BOARD #1

PATIENT	AGE	CHIEF COMPLAINT	VITAL SIGNS	PRIORITY	CHECKLIST
A	36 y/o woman	Vaginal bleeding	BP: 112/72 P: 86 R: 16 Temp: 37.2° C (98.9° F) Sat: 99% on room air	4	Last menstrual period (LMP) Pregnancy status? Quantify bleeding? Near syncope? Abdominal/Pelvic exam? POC Dx Urine HCG
B	6 y/o child	Head injury	BP: 104/65 P: 90 R: 18 Temp: 37.0° C (98.6° F) Sat: 99% on room air	6	Signs of AMS? LOC? Vomiting? Mechanism? Non-accidental trauma? Head exam? Neuro exam? POC Dx N/A
C	82 y/o man	Altered mental status	BP: 82/65 P: 125 R: 28 Temp: 39.1° C (102.3° F) Sat: 87% on room air	1	Awake Alert Oriented x? Airway? Breathing? Lung sounds? Pulses, distal edema? POC Dx EKG CXR Finger stick blood glucose
D	69 y/o woman	Chest pain	BP 150/76 P 82 R 16 Temp:36.6° C (97.9° F) Sat: 99% on room air	2	Character of chest pain? Radiation? Airway, lung sounds, pulses EKG CXR
E	44 y/o non-binary adult (assigned male at birth)	Headache	BP: 118/72 P: 78 R: 14 Temp: 36.9° C (98.4° F) Sat: 99% on room air	5	Description of Pain? (eg, Sudden onset? Quality/Intensity?) Neuro exam?
F	55 y/o woman	Abdominal pain	BP: 145/78 P: 98 R: 14 Temp: 37.7° C (99.9° F) Sat: 98% on room air	3	Quality/Intensity? Vomiting? Bowel Movement? Associated symptoms? Abdominal Exam? EKG

**Examiner:**

Here is your updated patient tracking board with additional patient information. Links to additional diagnostic studies are highlighted in gray for you to view. After reviewing the tracking board, let me know when you are ready to proceed.

*Show Tracking Board #2 and pause for Examinee response.*

- *IMPORTANT: Do NOT reveal stimuli until the Examinee has chosen to evaluate that specific patient (see the next Examiner prompt).*
- *It is possible that your Examinee may not go in the exact order of this script. That is ok - deviation from the optimal progression is accounted for in the Scoring Sheet. Just be sure to make note of the order and the choices that the Examinee makes.*
- *Addresses Objective 1:* ***Patient Prioritization and Initial Assessment***

**Table:** TRACKING BOARD #2

PATIENT	AGE	CHIEF COMPLAINT	VITAL SIGNS	CONDITION REPORT
A	36 y/o woman	Vaginal bleeding	BP: 112/72 P: 86 R: 16 Temp: 37.2° C (98.9° F) Sat: 99% on room air	LMP 6wks ago No near syncope or lightheadedness 4 pads in the last 24 hours Mild bilateral lower quadrant tenderness, R>L Urine HCG ordered
B	6 y/o child	Head injury	BP: 104/65 P: 90 R: 18 Temp: 37.0° C (98.6° F) Sat: 99% on room air	Witnessed fall from school playground equipment, 4 hours ago No loss of consciousness No vomiting Awake and alert Non-focal neuro exam No signs of basilar skull fracture
C	82 y/o man	Altered mental status	BP: 82/65 P: 125 R: 28 Temp: 39.1° C (102.3° F) Sat: 87% on room air	Lethargic Oriented to person, place but not time Diffuse rhonchi Non-focal neuro exam Blood sugar - 162 mg/dL ECG stimulus available CXR stimulus available
D	69 y/o woman	Chest pain	BP: 150/76 P: 82 R: 16 Temp: 36.6° C (97.9° F) Sat: 99% on room air	Substernal, Radiation to left arm Mild diaphoresis Lungs clear No leg swelling ECG ordered CXR ordered
E	44 y/o non-binary adult (assigned male at birth)	Headache	BP: 118/72 P: 78 R: 14 Temp: 36.9° C (98.4° F) Sat: 99% on room air	Gradual onset Similar to prior headaches Triggered by seasonal allergies Blurry vision (aura) Has had worse headaches Non-focal neuro exam
F	55 y/o woman	Abdominal pain	BP: 145/78 P: 98 R: 14 Temp: 37.7° C (99.9° F) Sat: 98% on room air	Severe, diffuse pain Actively vomiting Last BM 2 days ago, No flatus Abdomen diffusely tender High pitched bowel sounds

**Examiner:**

Based on what you know now, which patient do you want to treat first and what immediate stabilizing treatment would you provide?

- *Addresses Objective 2: ****Stabilization and Initial Management**** and Objective 5:* **Procedural Decision-Making & Execution**
- *Prompt to narrow down on Patient C.*
  ○ *Checklist for Patient C: Evaluate ABCDE*
  ○ *Airway implied to be patent/stable*
    ▪ *Apply O2, Nasal Cannula or Non-Rebreather*
    ▪ *30cc/kg crystalloid bolus*
    ▪ *Broad spectrum antibiotics*
    ▪ *Confirm allergies*
    ▪ *Order labs (Blood and Urine Culture)*
  ○ *NOW Reveal and Review Stimuli (ECG and CXR)*
    ▪ *Review ECG*^7^ *: Stimulus 1*
    ▪ *Review CXR*^8^ *: Stimulus 2*

*Pause for Examinee response.*

*Record answers on the* *Scoring Sheet.*

**Examiner:**

Thank you. Are there any tasks you would like to delegate to your care team to do while you're caring for this patient?

- *Addresses Objective 3:* ***Task Delegation and Team Management***
- *Remember, they have 2 nurses to help them; this prompt reminds the Examinee to deploy resources appropriately.*
  ○ *Delegation Checklist*
    ▪ *Assign RN to Patient D*
      - *IV, O2, Monitor, update VS*
      - *Obtain ECG and CXR results*
      - *Confirm allergies*
      - *Administer ASA 325mg PO*
    ▪ *Assign RN to Patient F*
      - *IV, O2, Monitor, update VS*
      - *Confirm allergies*
      - *Administer anti-emetic*
      - *Administer pain medication*

*Pause for Examinee response.*

*Record answers on the* *Scoring Sheet.*

**Examiner:**

You stabilized your first patient. Which patient would you choose to evaluate second and why?

- *Addresses Obj 1 (prioritize)*

*Pause for Examinee response.*

*Record answers on the* *Scoring Sheet.*

**Examiner:**

What immediate actions would you take?

- *Addresses Obj 2 (stabilize and manage)*
  ○ *Checklist for Patient D: Evaluate ABCDE*
    ▪ *Review ECG*^9^ *: Stimulus 3*
    ▪ *Review CXR*^10^ *: Stimulus 4*
    ▪ *Confirm allergies*
    ▪ *Administer ASA 325mg PO (if not already delegated)*
    ▪ *Administer NTG 0.4mg SL (treat pain)*
    ▪ *Order labs, including troponin*
    ▪ *Order heparin/anticoagulant*

*Pause for Examinee response.*

*Record answers on the* *Scoring Sheet.*

**Examiner:** Doctor, here are new results for Patient A.

*Examiner shows Stimulus 5. Urine Result.*

- *Addresses Obj 4:* ***Dynamic Adaptation & Task Switching***
  ○ *Task Switching Checklist:*
    ▪ *Confirm who is Patient A (36yo w vaginal bleeding)*
    ▪ *Stimulus 5 is a positive pregnancy test*
      - *Ask RN to update patient*
      - *Order labs including beta hcg quant and Type and Screen*
      - *Order pelvic Ultrasound*

*Pause for Examinee response.*

*Record answers on the* *Scoring Sheet.*

*Show Tracking Board #2.1 (updated showing Patient A as pregnant).*

**Table:** TRACKING BOARD #2.1

PATIENT	AGE	CHIEF COMPLAINT	VITAL SIGNS	CONDITION REPORT
A	36 y/o woman	Vaginal bleeding	BP: 112/72 P: 86 R: 16 Temp: 37.2° C (98.9° F) Sat: 99% on room air	Currently pregnant, G1P0 6wks by LMP 4 pads in the last 24 hours No OB visit or US yet Mild bilateral lower quadrant tenderness, R>L
B	6 y/o child	Head injury	BP: 104/65 P: 90 R: 18 Temp: 37.0° C (98.6° F) Sat: 99% on room air	Witnessed fall from school playground equipment, 4 hours ago No loss of consciousness, No vomiting Awake and alert Right scalp hematoma Non-focal neuro exam
C	82 y/o man	Altered mental status	BP: 82/65 P: 125 R: 28 Temp: 39.1° C (102.3° F) Sat: 87% on room air	Lethargic, Oriented to person, place but not time Diffuse rhonchi Non-focal neuro exam Blood sugar - 162 mg/dL ECG stimulus available CXR stimulus available
D	69 y/o woman	Chest pain	BP: 150/76 P: 82 R: 16 Temp: 36.6° C (97.9° F) Sat: 99% on room air	Substernal, Radiation to left arm Mild diaphoresis Lungs clear No leg swelling ECG ordered CXR ordered
E	44 y/o non-binary adult (assigned male at birth)	Headache	BP: 118/72 P: 78 R: 14 Temp: 36.9° C (98.4° F) Sat: 99% on room air	Gradual onset, Similar to prior headaches Triggered by seasonal allergies Blurry vision (aura) Has had worse headaches Non-focal neuro exam
F	55 y/o woman	Abdominal pain	BP: 145/78 P: 98 R: 14 Temp: 37.7° C (99.9° F) Sat: 98% on room air	Severe, diffuse pain Actively vomiting Last BM 2 days ago, No flatus Abdomen diffusely tender High pitched bowel sounds

**Examiner:**

You stabilized your second patient. Which patient would you choose to evaluate third and why?

- *Addresses Obj 1 (prioritize)*

*Pause for Examinee response.*

*Record answers on the* *Scoring Sheet.*

**Examiner:**

Thank you. What immediate actions would you take?

- *Addresses Obj 2 (stabilize and manage)*
  ○ *Checklist for Patient F: Evaluate ABCDE*
    ▪ *Administer anti-emetic and pain medication*
    ▪ *Administer crystalloid*
    ▪ *Order labs*
    ▪ *Order CT Abdomen Pelvis*

*Pause for Examinee response.*

*Record answers on the* *Scoring Sheet.*

**Examiner:**

Thank you. You've stabilized your third patient. Before you can evaluate the remaining patients, 2 additional Patients, G and H, arrive by EMS and have been added to your tracking board. Considering the remaining initial patients and the arrival of 2 new patients, who would you assess now and what immediate stabilizing treatments would you provide?

*Examiner shows Tracking Board #3.*

- *Addresses Obj 4 (Adapt and task switch), Obj 1 (prioritization), Obj 2 (stabilize and manage) and Obj 5 (procedures)*
- *Checklist for Patient H: Evaluate ABCDE*
- *Airway implied intact*
  ▪ *IV, O2, Monitor*
  ▪ *Recognize tension pneumothorax*
  ▪ *Perform needle decompression*
  ▪ *Reassess*
  ▪ *Administer local anesthesia and perform tube thoracostomy*

**Table:** TRACKING BOARD #3

PATIENT	AGE	CHIEF COMPLAINT	VITAL SIGNS	CONDITION REPORT
A	36 y/o woman	Vaginal bleeding	BP: 112/72 P: 86 R: 16 Temp: 37.2° C (98.9° F) Sat: 99% on room air	Currently pregnant, G1P0 6wks by LMP 4 pads in the last 24 hours No OB visit or US yet Mild bilateral lower quadrant tenderness, R>L
B	6 y/o child	Head injury	BP: 104/65 P: 90 R: 18 Temp: 37.0° C (98.6° F) Sat: 99% on room air	Witnessed fall from school playground equipment, 4 hours ago No loss of consciousness, No vomiting Awake and alert Right scalp hematoma Non-focal neuro exam
C	82 y/o man	Altered mental status	BP: 82/65 P: 125 R: 28 Temp: 39.1° C (102.3° F) Sat: 87% on room air	Lethargic, Oriented to person, place but not time Diffuse rhonchi Non-focal neuro exam Blood sugar - 162 mg/dL ECG stimulus available CXR stimulus available
D	69 y/o woman	Chest pain	BP: 150/76 P: 82 R: 16 Temp: 36.6° C (97.9° F) Sat: 99% on room air	Substernal, Radiation to left arm Mild diaphoresis Lungs clear No leg swelling ECG ordered CXR ordered
E	44 y/o non-binary adult (assigned male at birth)	Headache	BP: 118/72 P: 78 R: 14 Temp: 36.9° C (98.4° F) Sat: 99% on room air	Gradual onset, Similar to prior headaches Triggered by seasonal allergies Blurry vision (aura) Has had worse headaches Non-focal neuro exam
F	55 y/o woman	Abdominal pain	BP: 145/78 P: 98 R: 14 Temp: 37.7° C (99.9° F) Sat: 98% on room air	Severe, diffuse pain Actively vomiting Last BM 2 days ago, No flatus Abdomen diffusely tender High pitched bowel sounds CT Abdomen Pelvis ordered
G	22 y/o woman	Pain with urination	BP: 120/74 P: 84 R: 16 Temp: 37.2° C (98.9 °F) Sat: 99% on RA	Suprapubic pain, Subjective fever No flank pain, no hematuria, no vaginal discharge Sexually active with one male Suprapubic tenderness, no CVA tenderness
H	31 y/o man	Stab wound to chest	BP: 85/52 P: 134 R: 32 Temp: 36.9° C (98.4° F) Sat: 88% on RA	Stab wound to the left chest Speaking in 2-word sentences Short of breath with respiratory distress Absent breath sounds on the left

*Pause for Examinee response.*

*Record answers on the* *Scoring Sheet.*

**Examiner:**

You stabilized your fourth patient. Doctor, here are new results for Patient F.

*Examiner shows Stimulus 6. CT Abdomen Pelvis Report*

- *Addresses Obj 4:* ***Dynamic Adaptation & Task Switching and*** *Obj 5 (procedures)*
  ○ *Task Switching Checklist:*
    ▪ *Confirm who is Patient F (55yo w abdominal pain)*
    ▪ *Stimulus 6 confirms a Small Bowel Obstruction (SBO) on CT of abdomen and pelvis.*
      - *Update patient*
      - *Place Nasal Gastric Tube*
      - *Consult surgery*

*Pause for Examinee response.*

*Record answers on the* *Scoring Sheet.*

**Examiner:**

You stabilized your patient. Of the remaining unseen patients, in which order would you like to see them?

- *Addresses Obj 1 (prioritization)*
  ○ *Ideal priority*
    ▪ *Patient A (5)*
    ▪ *Patient E (6)*
    ▪ *Patient B (7)*
    ▪ *Patient G (8)*

**Examiner:**

All of your patients have been assessed and stabilized.

Thank you doctor, this concludes your case.

### Appendix 3: Prioritization: Run This Board Stimuli Slides and Stimuli to Print

#### Stimulus Inventory

**Table:**

**Candidate Task Sheet**		
**Tracking Board 1**		
**Tracking Board 2**		
**Tracking Borad 2.1**		
**Tracking Board 3**		
**#1**	**ECG**	**Patient C**
**#2**	**Chest X-ray**	**Patient C**
**#3**	**ECG**	**Patient D**
**#4**	**Chest X-ray**	**Patient D**
**#5**	**Urine Result**	**Patient A**
**#6**	**CT Abdomen Pelvis Report**	**Patient F**

**Table:** Prioritization Candidate Task Sheet

**CASE PARAMETERS**
This is a 15-minute case. You will interact with two examiners. This is an interview-style case without role playing. You will evaluate and treat multiple patients while ensuring those who require immediate care receive it as quickly as possible. You will have 2 nurses to complete orders and assist you in basic assessment of patients. You may face the arrival of additional patients, deterioration of existing patients, and workflow interruptions during the case.
**PATIENT INFORMATION**
Relevant information will be provided on your tracking board. Given multiple patients, you will be asked to identify individual features that would help you determine each patient’s acuity.
**TASK STATEMENT**
Your tasks are as follows: Given multiple patients, you will be asked to identify individual features that would help you determine each patient’s acuity. Identify the patients that most urgently require medical attention and provide stabilizing care. Effectively manage available clinical resources. Provide care in the setting of changing clinical conditions including new patients, changes in acuity, critical diagnostic results, and limitations of resources.

**Table:** TRACKING BOARD #1

PATIENT	AGE	CHIEF COMPLAINT	VITAL SIGNS
A	36 y/o woman	Vaginal bleeding	BP: 112/72 P: 86 R: 16 Temp: 37.2° C (98.9° F) Sat: 99% on room air
B	6 y/o child	Head injury	BP: 104/65 P: 90 R: 18 Temp: 37.0° C (98.6° F) Sat: 99% on room air
C	82 y/o man	Altered mental status	BP: 82/65 P: 125 R: 28 Temp: 39.1° C (102.3° F) Sat: 87% on room air
D	69 y/o woman	Chest pain	BP: 150/76 P: 82 R: 16 Temp: 36.6° C (97.9° F) Sat: 99% on room air
E	44 y/o non-binary adult (assigned male at birth)	Headache	BP: 118/72 P: 78 R: 14 Temp: 36.9° C (98.4° F) Sat: 99% on room air
F	55 y/o woman	Abdominal pain	BP: 145/78 P: 98 R: 14 Temp: 37.7° C (99.9° F) Sat: 98% on room air

**Table:** TRACKING BOARD #2

PATIENT	AGE	CHIEF COMPLAINT	VITAL SIGNS	CONDITION REPORT
A	36 y/o woman	Vaginal bleeding	BP: 112/72 P: 86 R: 16 Temp: 37.2° C (98.9° F) Sat: 99% on room air	LMP 6wks ago No near syncope or lightheadedness 4 pads in the last 24 hours Mild bilateral lower quadrant tenderness, R>L Urine HCG ordered
B	6 y/o child	Head injury	BP: 104/65 P: 90 R: 18 Temp: 37.0° C (98.6° F) Sat: 99% on room air	Witnessed fall from school playground equipment, 4 hours ago No loss of consciousness No vomiting Awake and alert Non-focal neuro exam No signs of basilar skull fracture
C	82 y/o man	Altered mental status	BP: 82/65 P: 125 R: 28 Temp: 39.1° C (102.3° F) Sat: 87% on room air	Lethargic Oriented to person, place but not time Diffuse rhonchi Non-focal neuro exam Blood sugar - 162 mg/dL ECG stimulus available CXR stimulus available
D	69 y/o woman	Chest pain	BP: 150/76 P: 82 R: 16 Temp: 36.6° C (97.9° F) Sat: 99% on room air	Substernal, Radiation to left arm Mild diaphoresis Lungs clear No leg swelling ECG ordered CXR ordered
E	44 y/o non-binary adult (assigned male at birth)	Headache	BP: 118/72 P: 78 R: 14 Temp: 36.9° C (98.4° F) Sat: 99% on room air	Gradual onset Similar to prior headaches Triggered by seasonal allergies Blurry vision (aura) Has had worse headaches Non-focal neuro exam
F	55 y/o woman	Abdominal pain	BP: 145/78 P: 98 R: 14 Temp: 37.7° C (99.9° F) Sat: 98% on room air	Severe, diffuse pain Actively vomiting Last BM 2 days ago, No flatus Abdomen diffusely tender High pitched bowel sounds

**Table:** TRACKING BOARD #2.1

PATIENT	AGE	CHIEF COMPLAINT	VITAL SIGNS	CONDITION REPORT
A	36 y/o woman	Vaginal bleeding	BP: 112/72 P: 86 R: 16 Temp: 37.2° C (98.9° F) Sat: 99% on room air	Currently pregnant, G1P0 6wks by LMP 4 pads in the last 24 hours No OB visit or US yet Mild bilateral lower quadrant tenderness, R>L
B	6 y/o child	Head injury	BP: 104/65 P: 90 R: 18 Temp: 37.0° C (98.6° F) Sat: 99% on room air	Witnessed fall from school playground equipment, 4 hours ago No loss of consciousness, No vomiting Awake and alert Right scalp hematoma Non-focal neuro exam
C	82 y/o man	Altered mental status	BP: 82/65 P: 125 R: 28 Temp: 39.1° C (102.3° F) Sat: 87% on room air	Lethargic, Oriented to person, place but not time Diffuse rhonchi Non-focal neuro exam Blood sugar - 162 mg/dL ECG stimulus available CXR stimulus available
D	69 y/o woman	Chest pain	BP: 150/76 P: 82 R: 16 Temp: 36.6° C (97.9° F) Sat: 99% on room air	Substernal, Radiation to left arm Mild diaphoresis Lungs clear No leg swelling ECG ordered CXR ordered
E	44 y/o non-binary adult (assigned male at birth)	Headache	BP: 118/72 P: 78 R: 14 Temp: 36.9° C (98.4° F) Sat: 99% on room air	Gradual onset, Similar to prior headaches Triggered by seasonal allergies Blurry vision (aura) Has had worse headaches Non-focal neuro exam
F	55 y/o woman	Abdominal pain	BP: 145/78 P: 98 R: 14 Temp: 37.7° C (99.9° F) Sat: 98% on room air	Severe, diffuse pain Actively vomiting Last BM 2 days ago, No flatus Abdomen diffusely tender High pitched bowel sounds

**Table:** TRACKING BOARD #3

PATIENT	AGE	CHIEF COMPLAINT	VITAL SIGNS	CONDITION REPORT
A	36 y/o woman	Vaginal bleeding	BP: 112/72 P: 86 R: 16 Temp: 37.2° C (98.9° F) Sat: 99% on room air	Currently pregnant, G1P0 6wks by LMP 4 pads in the last 24 hours No OB visit or US yet Mild bilateral lower quadrant tenderness, R>L
B	6 y/o child	Head injury	BP: 104/65 P: 90 R: 18 Temp: 37.0° C (98.6° F) Sat: 99% on room air	Witnessed fall from school playground equipment, 4 hours ago No loss of consciousness, No vomiting Awake and alert Right scalp hematoma Non-focal neuro exam
C	82 y/o man	Altered mental status	BP: 82/65 P: 125 R: 28 Temp: 39.1° C (102.3° F) Sat: 87% on room air	Lethargic, Oriented to person, place but not time Diffuse rhonchi Non-focal neuro exam Blood sugar - 162 mg/dL ECG stimulus available CXR stimulus available
D	69 y/o woman	Chest pain	BP: 150/76 P: 82 R: 16 Temp: 36.6° C (97.9° F) Sat: 99% on room air	Substernal, Radiation to left arm Mild diaphoresis Lungs clear No leg swelling ECG ordered CXR ordered
E	44 y/o non-binary adult (assigned male at birth)	Headache	BP: 118/72 P: 78 R: 14 Temp: 36.9° C (98.4° F) Sat: 99% on room air	Gradual onset, Similar to prior headaches Triggered by seasonal allergies Blurry vision (aura) Has had worse headaches Non-focal neuro exam
F	55 y/o woman	Abdominal pain	BP: 145/78 P: 98 R: 14 Temp: 37.7° C (99.9° F) Sat: 98% on room air	Severe, diffuse pain Actively vomiting Last BM 2 days ago, No flatus Abdomen diffusely tender High pitched bowel sounds CT Abdomen Pelvis ordered
G	22 y/o woman	Pain with urination	BP: 120/74 P: 84 R: 16 Temp: 37.2° C (98.9 °F) Sat: 99% on RA	Suprapubic pain, Subjective fever No flank pain, no hematuria, no vaginal discharge Sexually active with one male Suprapubic tenderness, no CVA tenderness
H	31 y/o man	Stab wound to chest	BP: 85/52 P: 134 R: 32 Temp: 36.9° C (98.4° F) Sat: 88% on RA	Stab wound to the left chest Speaking in 2-word sentences Short of breath with respiratory distress Absent breath sounds on the left

[Fig f4-jetem-10-5-ce312] [Fig f5-jetem-10-5-ce312] [Fig f6-jetem-10-5-ce312] [Fig f7-jetem-10-5-ce312]

**STIMULUS 5:** Urine Result Patient A

Urine HCG:	Positive

**STIMULUS 6:** CT Abdomen Pelvis Report Patient F

“… dilated, proximal small bowel loops with collapsed loops distally. Transition point in the RLQ with mesenteric stranding. Consistent with small bowel obstruction. Clinically correlate…”

### Appendix 5: Prioritization: Run This Board BLANK Tracking Board for Examinees to Print

**Table:** TRACKING BOARD - BLANK

PATIENT	AGE	CHIEF COMPLAINT	VITAL SIGNS	CONDITION REPORT
A				
B				
C				
D				
E				
F				
G				
H				

#### CERTIFYING EXAM ASSESSMENT

*Prioritization: Run This Board: Septic Shock, Acute Coronary Syndrome, Small Bowel Obstruction, and Penetrating Chest Trauma*

Learner: _________________________________________

### Appendix 6: Prioritization: Run This Board Scoring Sheet to Print

**Table:** PRIORITIZATION QUESTIONS (25%)

PATIENT	AGE	CHIEF COMPLAINT	CHECKLIST	Asked?	Total	Examinee
A	36y/o woman	Vaginal bleeding	**LMP, Pregnancy status?**		20	0
			**Quantify bleeding?**			
			**Urine HCG**			
			Abdominal/Pelvic exam?			
			Near syncope?		3	0
B	6 y/o child	Head injury	**Signs of altered mental status?**			
			**Mechanism?**			
			**Neuro exam?**			
			LOC? Vomiting?			
			Non-accidental trauma?			
			Head exam?		3	0
C	82 y/o man	Altered mental status	**AAOx?**			
			**Airway? Breathing?**			
			**EKG**			
			**CXR**			
			**Finger stick blood glucose?**			
			Lung sounds?			
			Pulses, distal edema?		5	0
D	69 y/o woman	Chest pain	**Characterization of chest pain**			
			**Radiation?**			
			**EKG**			
			**CXR**			
			Airway, Lung sounds, pulses		4	0
E	44 y/o non- binary adult (assigned male at birth)	Headache	**Description of Pain? (Eg, Sudden onset? Quality/Intensity?)**			
			**Neuro Exam?**		2	0
F	55 y/o woman	Abdominal Pain	**Quality/Intensity?**			
			**Vomiting?** **Bowel Movement?** **Associated Symptoms?**			
			**Abdominal Exam?**			
			EKG		3	0

**Table:** STABILIZATION ACTIONS & PROCEDURES (25%)

**PATIENT**	**AGE**	**CHIEF COMPLAINT**			**Total**	**Examinee**
C	82 y/o man	Altered mental status	**Apply O2, NC or NRB**	0	20.5	0
			**30cc/kg crystalloid bolus**	0		
			**Review ECG**	0		
			**Review CXR**	0		
			**Broad spectrum abx**	0		
			Confirm allergies	0		
			Order labs (BCx, UCx)	0	6	0
D	69 y/o woman	Chest pain	**Review ECG**	0		
			**Review CXR**	0		
			**Administer ASA 325mg PO (if not already delegated)**	0		
			**Administer NTG 0.4mg SL (treat pain)**	0		
			**Order labs, including troponin**	0		
			Confirm allergies	0		
			Order heparin/anticoagulant	0	6	0
F	55 y/o woman	Abdominal pain	**Administer anti-emetic**	0		
			**Administer pain medication**	0		
			**Order CT Abdomen/Pelvis**	0		
			Administer crystalloid	0		
			Order labs	0	4	0
H	31 y/o man	Stab wound to chest	**IV, O2, Monitor**	0		
			**Recognize tension pneumothorax**	0		
			**Perform needle decompression**	0		
			**Administer local anesthesia and perform tube thoracostomy**	0		
			Reassess	0	4.5	0

**Table:** DELEGATION ACTIONS (12.5%)

**PATIENT**	**AGE**	**CHIEF COMPLAINT**			**Total**	**Examinee**
D	69 y/o woman	Chest pain	**IV, O2, Monitor, update VS**	0	6	0
			**Obtain ECG and CXR results**	0		
			**Administer ASA 325mg PO**	0		
			Confirm allergies	0	3.5	0
F	55 y/o woman	Abdominal pain	**IV, O2, Monitor, update VS**	0		
			**Administer anti-emetic and pain medication**	0		
			Confirm allergies	0	2.5	0

**Table:** TASK SWITCHING ACTIONS (12.5%)

**PATIENT**	**AGE**	**CHIEF COMPLAINT**				**Total**	**Examinee**
A	36y/o woman	Vaginal bleeding	**Order labs incl beta hcg quant, type & screen, CBC**		0	5	0
			**Order pelvic US**		0		
			Ask RN to update patient		0	2.5	0
F	55 y/o woman	Abdominal pain	**Place NGT**		0		
			**Consult surgery**		0		
			Update patient		0	2.5	0
		**TOTAL**	65			51.5	0
			Yes	51.5			
			No	0			
			**TOTAL SCORE**	**0.00%**			
			**OVERALL SCORE**	**0.00%**			

**Table:** PRIORITIZATION ORDER (25%)

**PATIENT**	**AGE**	**CHIEF COMPLAINT**	**PATIENT**	**IDEAL ORDER**	**EXAMINEE ORDER**	**DISPLACEMENT**
A	36 y/o woman	Vaginal bleeding	A	5		5
B	6 y/o child	Head injury	B	7		7
C	82 y/o man	Altered mental status	C	1		1
D	69 y/o woman	Chest pain	D	2		2
E	44 y/o non-binary adult (assigned male at birth)	Headache	E	6		6
F	55 y/o woman	Abdominal pain	F	3		3
G	22 y/o woman	Pain with urination	G	8		8
H	31 y/o man	Stab wound to chest	H	4		4
				36 **TOTAL DISPLACEMENT** 36 **SCORE** 0 **PERCENTAGE** 0.00%		
			
			

### Appendix 7: Prioritization: Run This Board Debrief Guide

**Case Duration:** 15 minutes

**Debrief Duration:** 15–30 minutes

**Debrief Framework:** PEARLS (Promoting Excellence and Reflective Learning in Simulation)^6^

**Setting:** In-person or remote (Sim Zone 1 or 2)

**Tone:** Formative, reflective, coaching-oriented

#### Scenario Objectives

1. **Patient Prioritization & Initial Assessment:**
  - Analyze a patient tracking board and determine the most critical patients based on limited initial information.
  - Identify key history, physical exam findings, and immediate diagnostic tests needed for rapid decision-making.
2. **Stabilization & Initial Management:**
  - Implement appropriate immediate interventions for critically ill patients, such as noninvasive ventilation, fluid resuscitation, or procedural sedation.
  - Recognize and manage time-sensitive conditions, including airway compromise, hemodynamic instability, and altered mental status.
3. **Task Delegation & Team Management:**
  - Identify tasks that can be delegated to the healthcare team to optimize workflow and efficiency.
  - Communicate clear and concise instructions to supporting staff during patient management.
4. **Dynamic Adaptation & Task Switching:**
  - Adjust clinical priorities based on additional diagnostic data and evolving patient conditions.
  - Reprioritize care as new patients arrive, balancing initial assessments with ongoing interventions.
5. **Procedural Decision-Making & Execution:**
  - Determine the appropriate use of sedation and procedural interventions for acutely ill patients.
  - Perform or direct critical procedures such as intubation, fracture reduction, and vascular access placement.
6. **Case Completion & Reflection (Debrief):**
  - Review and reflect on decision-making strategies at the conclusion of the case.
  - Identify areas for improvement in prioritization, delegation, and procedural execution.

##### I. Reactions (2–3 min)

*Goal: Allow the learner to process emotional and cognitive responses.*

- "How did that feel overall?"
- "What was your immediate reaction to seeing all the patients on the tracking board?"
- "Were there any moments you felt particularly confident or overwhelmed?"

##### II. Description (3–5 min)

*Goal: Establish a shared understanding of what happened.*

- "Walk me through how you approached the initial board."
- "What was your rationale for choosing your first patient?"
- "How did you adapt as new patients or information came in?"

##### III. Additional Scoring Rubric Information

***Skip this section if you are using the automatic Scoring Sheet Scoring Rubic***

For Prioritization Questions scoring:

**Table:**

• Count the number of “y” marks for each patient.	
○ Patient A	
▪ If > 3, score 3	______
▪ If < 3, score the counted number (eg, 2, 1, or 0)	
○ Patient B	
▪ If > 3, score 3	______
• Count the number of “y” marks for each patient.	
○ Patient A	
▪ If > 3, score 3	______
▪ If < 3, score the counted number (eg, 2, 1, or 0)	
○ Patient B	
▪ If > 3, score 3	______
▪ If < 3, score the counted number	
○ Patient C	
▪ If > 5, score 5	______
▪ If < 5, score the counted number	
○ Patient D	
▪ If > 4, score 4	______
▪ If < 4, score the counted number	
○ Patient E	
▪ If > 2, score	______
▪ If < 2, score the counted number	
○ Patient F	
▪ If > 3, score 3	______
▪ If < 3, score the counted number	
○ **Prioritization Question Raw Total**	______

For Stabilization Actions and Procedures, Delegation Actions and Task switching, **bolded items receive 1 point.** Unbolded items receive 0.5 points.

For Prioritization Order, compare the Examinee’s order to the Ideal Order. Subtract Examinee value from the Ideal Order value and take the absolute value. Add all absolute values for the total displacement and apply this equation:

*Prioritization Order Raw Total = (36-total displacement)/36*

*As a reminder, the total score is as follows from the Score Sheet:*

*Total Score = Prioritization Questions Weighted Score + Stabilization and Initial Management Questions Weighted Score + Delegation Tasks Weighted Score + Task Switching Weighted Score + Prioritization Order Weighted Score*

*Total Score = ((___/20)*0.25)+((___/20.5)*0.25)+((___/6)*0.125)+((___/5)*0.125)+(((36-total displacement)/36)*0.25).*

##### IV. Analysis (20 min)

*Goal: Facilitate reflection and provide structured feedback based on rubric scores.*

*Based on our beta testing, a passing score is probably a Total Score = 70–75%*

##### Domain 1: Patient Prioritization Questions (25%)

**Raw Score:** _____ *(from cell H4)* **/ 20**

**Percentage:** _____ *(from cell I4)*

*Objective 1: Patient Prioritization & Initial Assessment:*

- Analyze a patient tracking board and determine the most critical patients based on limited initial information.
- Identify key history, physical exam findings, and immediate diagnostic tests needed for rapid decision-making

**Facilitator Prompts:**

- "How did you decide what key questions to ask when initially assessing patients?"
- "What strategies do you use to ensure you don’t miss red flags with limited information?"

**Facilitator Notes:**

- Highlight early screening for critical info (eg, mental status, pregnancy status, blood sugar).
- Identify patterns in what was missed or well-covered.

##### Domain 2: Stabilization & Initial Management (25%)

**Raw Score:** _____ *(from cell H35)* **/ 20.5**

**Percentage:** _____ *(from cell I35)*

*Objective 2: Stabilization & Initial Management:*

- Implement appropriate immediate interventions for critically ill patients, such as noninvasive ventilation, fluid resuscitation, or procedural sedation.
- Recognize and manage time-sensitive conditions, including airway compromise, hemodynamic instability, and altered mental status.

**Objective 5: Procedural Decision-Making & Execution:**

- Determine the appropriate use of sedation and procedural interventions for acutely ill patients.
- Perform or direct critical procedures such as intubation, fracture reduction, and vascular access placement.

**Facilitator Prompts:**

- "Let’s talk about your immediate actions for your first few patients. What went well?"
- "Were there any moments where you felt stuck on what the next step should be?"

**Facilitator Notes:**

- Reinforce the importance of C-ABCDE, “IV, O2, Monitor” framework.
- Discuss time-sensitive interventions like fluids, oxygen, ASA, antibiotics.

##### Domain 3: Task Delegation & Team Management (12.5%)

**Raw Score:** _____ *(from cell H61)* **/ 6**

**Percentage:** _____ *(from cell I61)*

*Objective 3: Task Delegation & Team Management:*

- Identify tasks that can be delegated to the healthcare team to optimize workflow and efficiency.
- Communicate clear and concise instructions to supporting staff during patient management.

**Facilitator Prompts:**

- "How did you decide when and what to delegate to the nurses?"
- "Were there tasks you could have handed off earlier or more clearly?"

**Facilitator Notes:**

- Look for use of parallel processing and resource optimization.
- Assess clarity of communication.

##### Domain 4: Task Switching & Adaptation (12.5%)

**Raw Score:** _____ *(from cell H70)* **/ 5**

**Percentage:** _____ *(from cell I70)*

*Objective 4: Dynamic Adaptation & Task Switching:*

- Adjust clinical priorities based on additional diagnostic data and evolving patient conditions.
- Reprioritize care as new patients arrive, balancing initial assessments with ongoing interventions.

**Facilitator Prompts:**

- "What changed when you received new data like a positive HCG or CT findings?"
- "How did you manage juggling competing priorities?"

**Facilitator Notes:**

- Highlight cognitive flexibility and real-time reprioritization.
- Emphasize safe handoffs and info integration.

##### Domain 5: Prioritization Order (25%)

**Percentage** *(from G13)***: ((36-_____*****absolute total displacement*****)/36)**

*Objective 1: Patient Prioritization & Initial Assessment:*

- Analyze a patient tracking board and determine the most critical patients based on limited initial information.
- Identify key history, physical exam findings, and immediate diagnostic tests needed for rapid decision-making.

**Facilitator Prompts:**

- "Let’s look at your final prioritization order. How did you determine who to see next?"
- "Would you reorder any patients if you had a chance to redo the case?"

**Facilitator Notes:**

- Use the displacement score as objective data.
- Discuss balance between acuity, available data, and cognitive load.

##### V. Summary & Takeaways (3–5 min)

- "What’s one thing you feel you did well today?"
- "What’s one thing you want to work on before your next high-stakes simulation or real shift?"

**Facilitator Notes:**

- Offer targeted praise and one focused growth point.
- Connect simulation takeaways to clinical ED performance.

**Table:** Blank Scoring Table Notes

Domain	Score	Strengths	Growth Opportunities
Patient Prioritization Questions	___ / 20 * 0.25		
Stabilization & Initial Management	___ / 20.5 * 0.25		
Task Delegation & Team Management	___ / 6 * 0.125		
Task Switching & Adaptation	___ / 5 * 0.125		
Prioritization Order	((36- ___ /36) * 0.25		
**Total %**	_________		
